# Medical students’ perception of the role of nursing in medical care: implications for interprofessional education

**DOI:** 10.3389/fmed.2026.1877755

**Published:** 2026-08-10

**Authors:** Lukas Valerius, Jonathan Hagedorn, Christian Albert, Johann Wendler, Katrin Werwick, Rüdiger C. Braun-Dullaeus, Philipp Stieger

**Affiliations:** 1 University Clinic for Cardiology and Angiology, Otto-von-Guericke University Magdeburg, Magdeburg, Germany; 2 Department of Hematology, Oncology and Palliative Medicine, Klinikum Magdeburg, Magdeburg, Germany; 3 Department of Nephrology, Central Clinic Bad Berka, Bad Berka, Germany; 4 University Clinic for Urology, Urooncology, Robot-Assisted and Focal Therapy, University Hospital Magdeburg A.ö.R., Medical Faculty of the Otto-von-Guericke-University Magdeburg, Magdeburg, Germany; 5 Department of Strategic and Corporate Development, University Clinic Magdeburg, Magdeburg, Germany

**Keywords:** clinical placements, interprofessional collaboration, medical education, medical students, nursing roles, professional identity formation

## Abstract

**Introduction:**

Interprofessional collaboration between nurses and physicians is essential for effective patient care, safe clinical workflows, and patient-centered outcomes. Despite its importance, interprofessional knowledge is insufficiently integrated into German medical education, potentially limiting students’ readiness for collaborative practice and their understanding of professional roles in clinical settings. This study explored how medical students perceive nurses’ work, nursing-related learning experiences, and interprofessional collaboration and Role Understanding within undergraduate medical education.

**Methods:**

Fourteen medical students (1st–6th year) at the University of Magdeburg participated in semi-structured interviews recruited using purposive sampling to ensure variation across study years. Transcripts were analyzed using Mayring’s qualitative content analysis to identify categories and themes. Analytical rigor was ensured through independent coding and interdisciplinary validation in a research workshop.

**Results:**

Seven themes were identified: Interprofessional Collaboration and Role Understanding, First Clinical Experiences, Nursing During the Practicum, Nursing-Related Learning Experiences During Medical School, Desired Nursing-Related Learning Experiences, Importance of Nursing, and Patient Well-being. The mandatory nursing practicum emerged as a key formative experience shaping early interprofessional role understanding. Students reported variability in supervision and structure, alongside limited curricular integration of nursing content. While interprofessional collaboration was considered essential, participants expressed uncertainty regarding nursing roles and responsibilities and emphasized the need for structured opportunities for interprofessional and nursing-related learning, including clinical skills, communication, and management of complex patient situations.

**Conclusion:**

Medical students’ perceptions of nursing are strongly shaped by early and variably structured clinical experiences, particularly the nursing internship. The findings suggest that current medical curricula insufficiently support the systematic development of interprofessional role understanding. The structured integration of nursing-related learning experiences and explicit teaching about nursing roles, nursing-related practical skills and clinical workflows into medical education may strengthen interprofessional collaboration and support patient-centered care.

## Introduction

Effective patient care in the hospital setting relies on interprofessional collaboration between physicians and nursing staff, who represent the two largest groups within the healthcare workforce. Accordingly, successful teamwork requires a clear understanding and appreciation of professional roles and responsibilities among these professions (1, 2).

Gaps in interprofessional understanding and communication may be rooted in insufficient mutual recognition of professional roles (3). Such misconceptions may arise from early encounters between future physicians and nursing staff during Medical School. Clear learning objectives regarding interprofessional competencies have been integrated into some medical education frameworks. A comparison of the German National Competence-Based Learning Objectives Catalog for Medicine (NKLM 2.0) (4) with the Canadian CanMEDS Role of a Collaborator (5) underscores the need to systematically integrate these competencies into undergraduate medical training.

Interprofessional education (IPE) is widely regarded as a key strategy for preparing healthcare professionals for collaborative practice. According to the World Health Organization and the Center for the Advancement of Interprofessional Education (CAIPE), IPE occurs when learners from different professions learn with, from, and about each other to improve collaboration and the quality of care (1, 6). Beyond the acquisition of collaborative skills, IPE aims to foster role clarification, mutual respect, shared decision-making, and a better understanding of professional responsibilities. Role clarification is considered a central mechanism through which IPE contributes to effective teamwork and patient-centered care.

In contrast to these internationally recognized educational objectives, German medical students frequently report limited or stereotypical views of the nursing profession, which may contribute to misconceptions and communication barriers that negatively impact teamwork (7). Despite increasing policy attention to interprofessional education in Germany, empirical research examining medical students’ experiences with nursing remains limited, and qualitative evidence exploring how students perceive nursing roles, nursing-related learning experiences, and interprofessional collaboration is particularly scarce. The importance of interprofessional education and collaborative competencies has been repeatedly emphasized by policy-makers and explicitly addressed within the German Medical Licensure Act (8, 9). However, despite this acknowledgment, mandatory nursing internships for German medical students, which span up to 3 months, lack clearly defined curricular learning objectives and structured reflection.

These internships, however, are often unstructured and may provide limited opportunities for systematic learning about nursing roles, nursing practices, and nursing-related learning experiences. Consequently it remains insufficiently understood how medical students integrate these experiences into their emerging understanding of clinical practice during early medical training. Within structured yet often unstandardized learning formats such as the mandatory nursing internship in German medical education, these experiences may be perceived as either supplementary to or constitutive of students’ emerging clinical competence.

This study is based on the premise that understanding nursing-related practices constitutes a foundational element of physicians’ emerging understanding of clinical and interprofessional practice.

The aim of this study was to explore how German medical students perceive nursing roles, nursing-related learning experiences, and interprofessional collaboration within undergraduate medical education.

## Materials and methods

### Study design and context

This qualitative study used semi-structured interviews to explore medical students’ perceptions of nursing and interprofessional collaboration in clinical training. A qualitative descriptive design with an inductive approach was chosen to capture participants’ subjective experiences and meanings. The study was conducted within the framework of the University of Magdeburg’s LEMMING project (“Interprofessionelle Lehre in den Praxisphasen des Medizinstudiums am Universitätsklinikum Magdeburg”), an educational initiative aimed at strengthening interprofessional competencies in undergraduate medical training. Although embedded within a single institutional project, participants were recruited from multiple German medical schools to capture perspectives across different educational contexts. Germany provides a particularly relevant context for this study, as the mandatory nursing internship represents a standardized yet variably implemented form of early clinical exposure, offering a unique opportunity to examine how structured but insufficiently formalized interprofessional learning environments shape medical students’ professional role formation.

### Study context

Medical training in Germany comprises a preclinical and a clinical phase and includes a mandatory 3-months nursing internship (Pflegepraktikum), which is typically completed during the early stages of medical training. Admission to German medical schools is highly competitive and primarily based on secondary school achievement alongside additional university-specific selection criteria.

### Participants and recruitment

Recruitment followed a peer-to-peer approach. Two medical students from different academic years (LV and JH) conducted interviews with fellow students from multiple cohorts and several German medical schools. Participation was voluntary. To facilitate representation across different stages of medical training, students were purposively invited to ensure diversity regarding gender and training stage.

Participants were recruited from multiple German medical faculties; however, no formal stratification by institution was performed. The final sample included students across all years of study (years 1–6), enabling the inclusion of perspectives from different stages of professional development. Sampling was continued until thematic sufficiency was achieved, defined as the point at which no substantially new categories or themes emerged from consecutive interviews. The research team judged thematic sufficiency to have been reached after approximately 12 interviews, with the remaining interviews primarily confirming existing categories.

### Research team and reflexivity

The interviews and initial coding were conducted by two medical students (LV, JH) who were themselves part of the target population. This peer-to-peer constellation may have facilitated open communication but also entails a risk of social desirability bias and shared assumptions. To address this, interviewers followed a structured interview guide, received prior training in qualitative interviewing, and explicitly emphasized voluntariness and anonymity to participants.

Throughout the research process, reflexive discussions were conducted within the research team to critically examine potential preconceptions and their influence on data collection and analysis. The researchers’ own experiences as medical students may have influenced both the salience of certain topics during data collection and the interpretation of participants’ accounts during analysis. To mitigate this potential bias, emerging interpretations were regularly discussed within the wider interdisciplinary research team. These reflexive processes were complemented by interdisciplinary discussions during a research workshop, which provided perspectives beyond the immediate research team and helped to critically challenge shared assumptions and preliminary interpretations.

### Data collection

Data were collected between September 2024 and March 2025. Interviews were conducted in German using a semi-structured interview guide developed *de novo* based on a focused literature review on interprofessional education and nursing content in medical training (Supplementary Material 1). The guide comprised seven anchor questions addressing perceptions of nursing roles, nursing-related learning experiences, the relevance of interprofessional collaboration, and reflections on work-based learning. The guide was piloted and slightly refined prior to data collection.

Interviews were conducted either in person or via video conferencing, depending on availability and local conditions. Interviews lasted approximately 30–45 min. Field notes documenting contextual aspects of the interview situation were recorded. All interviews were audio-recorded, transcribed verbatim, and anonymized according to Przyborski (10). Each transcript received an anonymized code for citation.

Data collection and preliminary analysis were conducted iteratively, allowing emerging insights to inform subsequent interviews in line with qualitative research principles. Following every two to three interviews, transcripts were reviewed within the research team and preliminary categories were discussed. Emerging topics identified during these reviews were explored in greater depth through follow-up probes in subsequent interviews where appropriate.

### Data analysis

A qualitative inductive content analysis following Mayring’s approach was conducted (11). Analytical rigor was ensured through independent coding, iterative refinement of the category system, consensus-based resolution of coding discrepancies, and investigator triangulation.

After anonymization, transcripts were read to define units of analysis. The unit of analysis consisted of meaning units representing coherent statements or passages related to students’ perceptions of nursing roles, nursing-related learning experiences, and interprofessional collaboration. Following Mayring’s inductive category development approach, relevant text passages were coded, paraphrased, generalized to a higher level of abstraction, and iteratively reduced into category definitions. Categories were continuously revised through comparison with subsequent material until a stable category system was established. Preliminary coding began during the data collection phase and continued alongside ongoing recruitment and interviewing. This iterative process supported refinement of the category system and assessment of thematic sufficiency across the dataset.

Coding was conducted independently by two researchers (LV, JH), followed by consensus discussions to resolve coding discrepancies and iteratively refine the category system through constant comparison across transcripts.

Differences in coding were discussed and resolved through consensus, and the category system was continuously refined.

To enhance analytical rigor, the coding process involved multiple iterative cycles and constant comparison across transcripts. The final category system was applied to the complete dataset and checked for internal consistency.

Internal communicative validation and ongoing assessment of thematic sufficiency were performed within the research team. The category system was refined through iterative discussion and validated against the remaining data. In addition, key categories and representative excerpts were reviewed in an interdisciplinary research workshop involving researchers with expertise in medical education and qualitative methodology. This process served as investigator triangulation and external validation, challenging preliminary interpretations and refining the category system.

Participant validation (member checking) was not performed due to logistical constraints and the anonymized nature of the data. To mitigate this, analytical rigor was strengthened through investigator triangulation, independent coding, and iterative category development. Professional Identity Formation (PIF) and Extended Professional Identity Theory (EPIT) were not used as an *a priori* analytical framework or to guide the coding process. Instead, these theories were applied during the interpretation of the findings after the inductively derived category system had been established, thereby supporting the discussion of the results without influencing category development.

Qualitative data management and coding were conducted using Microsoft Excel (version 16.89, Redmond, Washington, USA). Given the relatively small dataset, Excel supported transparent organization and iterative comparison. It enabled transparent structuring of codes, categories, and coding iterations, thereby facilitating an audit trail of analytical decisions. Consistent with qualitative methodological standards, analytical rigor was ensured through the systematic coding process rather than reliance on specific software tools.

**FIGURE 1:**
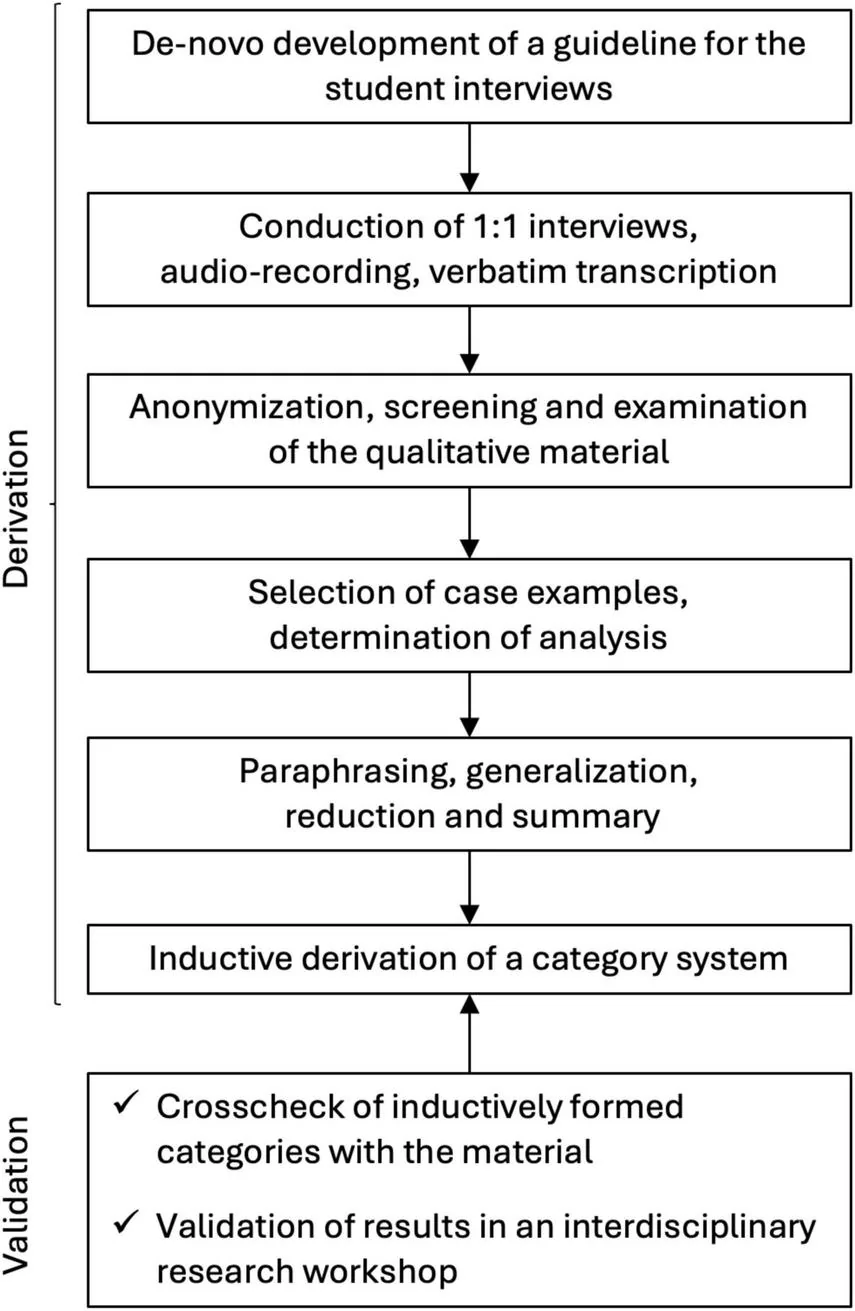
Study flow chart.

The methodological study flowchart is shown in Figure 1.

### Translation of excerpts

Interview excerpts presented in the results were translated into English using forward–backward translation to ensure semantic accuracy.

### Ethics and reporting

The study adhered to the Declaration of Helsinki and was approved by the Otto-von-Guericke University Medical Faculty Ethics Committee, Magdeburg (Case No. 48/25). Written informed consent was obtained from all participants. Reporting followed the COREQ checklist (12).

## Results

The final sample consisted of 14 participants (8 females, 6 males) with a mean age of 24.57 years (SD = 2.44, range 20–28). The median year of study was year 4 (range = 1–6). Participants were recruited from multiple German medical schools; however, the majority were enrolled at the University of Magdeburg (*n* = 11), while three participants studied at other institutions (Lübeck, Heidelberg, and Hamburg), reflecting the recruitment base of a single institutional educational initiative, with most participants drawn from the host site and limited external representation.

### Main categories and themes resulting from qualitative content analysis

Seven main categories were identified from the material: “Interprofessional Collaboration and Role Understanding,” “First Clinical Experiences,” “Nursing During the Practicum,” “Nursing-Related Learning Experiences During Medical School,” “Desired Nursing-related Learning Experiences,” “Importance of Nursing” and “Patient Well-being.” Additional subthemes derived from the material can be found in Table 1. Underlying anchor quotations are available in Supplementary Material 2.

**TABLE 1:** Categories and themes derived from the qualitative content analysis.

1. Interprofessional collaboration and role understanding
1.1 Communication and understanding between the professions
1.2 Interprofessional cooperation: perceived attitude and respect felt
1.3 Experiences working together with nursing staff in everyday clinical practice
1.4 Structural aspects of interprofessional collaboration
1.5 Individually ascribed meaning of interprofessional collaboration
2. First clinical experiences
2.1 First practical experiences and contact with daily clinical practice
2.2 Subjective experience of the nursing practicum
2.3 Structure and preparation of the nursing practicum
2.4 Patient contact and practical activities in the context of nursing
3. Nursing during the practicum
3.1 Personal perception of nursing as a professional group in the context of the practicum
3.2 Supervision, guidance, and mentoring by nursing staff
3.3 Structure and organization of the nursing practicum
4. Nursing-related learning experiences during Medical School
4.1 Curricular integration of nursing-related learning opportunities
4.2 Theoretical learning about nursing
4.3 Practical nursing-related learning during Medical School
4.4 Perception of the importance of nursing-related content during Medical School
5. Desired nursing-related learning experiences
5.1 Practical skills and workflows
5.2 Patient interaction and communication skills
5.3 Dealing with special patient groups and stressful situations
5.4 Nursing-related foundational knowledge
6. Importance of nursing
6.1 Personal significance
6.2 Systemic significance
6.3 Change in the perception of nursing
7. Patient well-being
7.1 Teamwork and patient well-being
7.1 7.2 Patient-centered care

#### Interprofessional collaboration and role understanding

The following subcategories emerged:

- Communication and understanding between the professions
- Interprofessional cooperation: Perceived attitude and respect felt
- Experiences working together with nursing staff in everyday clinical practice
- Structural aspects of interprofessional collaboration
- Individually ascribed meaning of interprofessional collaboration

Students highlighted the essential role of interprofessional collaboration in patient care, noting that understanding nursing roles underpins teamwork. Suboptimal communication led to misunderstandings and missed cooperation opportunities.

“*OK, well, in principle, one must understand what the other working groups do all day long and where their areas of responsibility lie. […], communication is something one also has to learn in a somewhat practical way, and you can also gladly look at all sides of it.” 1CJYR*

Several interviewees expressed that nurses are indispensable in hospital routines, yet are frequently undervalued in everyday interactions.

*“Well, without nursing, nothing would really work in the hospital, and yes, I also find that nursing is always underappreciated there, for example.” UR6P1*

Students described situations in which collaboration with nurses was suboptimal, reinforcing the need for more structured opportunities to learn about nursing tasks and perspectives during medical training.

Participants noted that many basic clinical skills are performed by nurses but later expected of medical students, highlighting structural challenges and the need for clearer curricular integration. Interprofessional collaboration was viewed as a key competence for effective teamwork and quality care.

*“OK, so I think it’s really important because collegial cooperation can only work if you also have some knowledge of it and also get involved in that area, especially when you have contact during a practicum or something like that.” JXJ1N*

#### First clinical experiences

Students frequently referred to the nursing internship as their first meaningful exposure to clinical routines. Within this category, the following subcategories emerged:

- First practical experiences and contact with daily clinical practice
- Subjective experience of the nursing practicum
- Structure and preparation of the nursing practicum
- Patient contact and practical activities in the context of nursing

Students described the nursing internship as their initial point of contact with clinical practice and patient care. For many, this represented the first opportunity to experience the daily routines of a hospital environment and to interact directly with patients.

*“So if you have never been to a hospital before, or worked in the medical field in general, that’s where you really first learn the basics. Thus, you learn how to deal with people, how to talk to people, how to talk to patients, how to deal with patient histories, and you also come into contact for the first time with sick people or care-dependent people.” X9T3B*

These encounters were often formative, shaping early impressions of teamwork and patient management. Experiences varied widely: some students reported meaningful integration into ward routines and supportive staff interactions, whereas others felt confined to menial tasks (e.g., cleaning or bed-making) with limited educational value.

*“And unfortunately, it often came down somehow to cleaning and making beds and so on. These low-level tasks didn’t add any value for me.” 20VFF*

Organizational challenges included lack of structured preparation, leaving students underutilized. Orientation sessions and clearer learning objectives were suggested.

*“Very exhausting. So, I did not feel very appreciated as I found 3 months of unpaid work during the holidays a lot. So, I think less would have been enough, and more appreciative interactions in return.” GPR6C*

Suggestions included prior orientation sessions and clearer guidance regarding learning objectives to enhance the educational benefit of the experience.

Despite these difficulties, students emphasized the value of patient contact. Engaging in basic care, assisting with ward routines, and observing nurse–patient interactions provided insights into communication and empathy. These experiences offered an entry point into hospital life, with learning outcomes depending on supervision and support.

#### Nursing during the practicum

Students highlighted a wide range of experiences during their mandatory nursing practicum, often describing the rotation as both a challenge and an opportunity to gain practical insights. Within this category, the following subcategories emerged:

- Personal perception of nursing as a professional group in the context of the practicum
- Supervision, guidance, and mentoring by nursing staff
- Structure and organization of the nursing practicum

Several participants considered the internship essential for understanding the nursing role and emphasized the importance of contact with nurses and recognition of their expertise.

*“I found, for example, that where I really noticed this the first time was naturally during the nursing practicum, which is sort of the first thing you experience when you start your studies. I think it’s super important to have contacts there, simply to get to know the other side a bit at first. Also, the appreciation that is shown to nursing staff. And what also really struck me was that the nurses naturally see patients for much longer than the doctors do. And I have very often noticed that there were moments when I thought to myself afterwards that one should have listened to the nurse sooner or asked earlier, then one could have- I don’t know, prevented this and that.” BJ1V6*

Others stressed that their impressions depended on the degree of staff supervision and engagement, reporting both supportive guidance and situations in which they performed routine tasks without sufficient explanation.

*“The collaboration was actually really nice with both, they showed a lot, I was actually always involved in many things.” UR6P1*

*“So not much in terms of specialized skills, a bit like everyday life on the ward. I just did all the work that no one else wanted to do.” GPR6C*

Students also noted organizational issues, including lack of structured orientation and clear learning goals, which limited the internship’s educational value. Many reported that observing nurses’ daily responsibilities fostered appreciation for their essential role. The internship was formative but highly variable, with outcomes depending on the ward environment and staff willingness to integrate students.

#### Nursing-related learning experiences during Medical School

Within this category, the following subcategories emerged:

- Curricular integration of nursing content
- Theoretical learning about nursing
- Practical nursing-related learning during Medical School
- Perception of the importance of nursing content during the studies

Students predominantly criticized the integration of nursing-related learning content in the medical curriculum. Nursing was seen as underrepresented, leaving students uncertain about nurses’ roles and responsibilities. One student noted that even after the 90-days internship, fundamental questions about nursing responsibilities persisted.

*“Well, I think that medical school teaches you very little about nursing and how to care for patients properly. One has a 90-day nursing practicum in the pre-clinical phase; nevertheless, many uncertainties remain […] And these are also points that are definitely being addressed more and more thoroughly in nursing training in comparison to medical school.” POA6W*

Opinions diverged on the extent of theoretical knowledge needed. Some argued that not every detail of nursing care, such as patient washing, is necessary, but basics like patient positioning or safe mobilization are valuable, as these situations may arise unexpectedly.

*“So in terms of theoretical content, I do not necessarily need to know how to wash a patient, I agree with that, I don’t necessarily need that, but maybe a couple of little things, like how to position the patient properly, how to get patients out of bed properly without their sliding halfway down somewhere, because you sometimes have to do that yourself. Sometimes you find yourself in situations where you just have to know these things. And I am of the opinion that’s something you should already know.” 23OF9*

Experiences also differed regarding practical content. Some participants found minimal engagement sufficient, while others criticized the lack of opportunities to learn basic skills, such as setting up an infusion or handling an intravenous line, suggesting their integration into structured teaching to better prepare students for clinical responsibilities.

*“I think that I’m still lacking some very basic skills that nursing staff have, for example, like how to set up an infusion correctly, how to handle a peripheral IV line properly so that it works well and runs smoothly. I didn’t learn that during the nursing practicum. […] But I think it would also somehow be helpful to have an afternoon again or maybe even a block practicum, you know, to integrate that.” 20VFF*

Finally, several students highlighted the broader importance of nursing in medical education, linking it to collegial collaboration and effective interprofessional teamwork. Others considered the current scope adequate and saw no need for additional nursing content.

#### Desired nursing-related learning experiences

Students articulated a set of specific expectations that can be grouped into the following subcategories:

- Practical skills and workflows
- Patient interaction and communication skills
- Dealing with special patient groups and stressful situations
- Nursing-related knowledge and learning content

Students expressed clear expectations for nursing content in the curriculum, most frequently requesting practical skills such as infusion management, wound care, and safe handling of medical devices.

*“I think that a precise, structured way of working [is important] – like, how do I organize certain procedures? How do I ensure the hygienic execution of specific nursing routines?” POA6W*

These skills were considered essential for daily clinical practice and are often expected of medical students without formal instruction.

Participants also emphasized the importance of communicating effectively with patients and relatives, highlighting empathy, respect, and clarity as crucial for building trust and ensuring patient well-being.

*“And also, if I understood correctly, simply the contact with patients in general.” POA6W*

Several students noted the need for preparation in handling challenging situations and vulnerable patients, such as those with dementia, children, or in highly stressful conditions. These encounters were described as demanding and requiring strategies insufficiently addressed in current teaching.

*“And also what are some conflict situations that can arise in everyday life with patients? How do I deal with certain patient groups where it’s difficult to follow the usual nursing routine, for example, in cases of dementia, or if patients could potentially be aggressive, or when they can’t engage, or even when they’re not capable of giving consent? And how do you handle this, so that even the more difficult cases are taken into consideration, also these conflict resolution skills [are needed]!” POA6W*

Beyond communication with patients, several participants also emphasized the need to develop communication skills within the interprofessional team. Students highlighted the importance of understanding how information should be exchanged between nurses and physicians to support safe and coordinated patient care. As one participant explained:

*“How can I facilitate communication between nursing and doctors? What can I personally do so that no information is lost and the patient is treated best in the end?”* 23OF9

#### Importance of nursing

Students addressed the significance of nursing on multiple levels, which can be grouped into the following subcategories:

- Personal significance
- Systemic significance
- Change in the perception of nursing

Students reflected on the relevance of nursing for themselves and the healthcare system. Several emphasized that nursing involves close patient contact, fostering empathy, and described these experiences as opportunities to gain a deeper understanding of patient needs beyond medical knowledge.

*“I actually think nursing is quite important, within the framework of the studies, for teaching these basics like I just said, but also nursing imparts somehow an element of human closeness, I would simply say. So, there’s also a big part of empathy involved. Obviously, you don’t learn all the medical stuff, like the concrete medical things, but you do learn, or you just simply somehow develop there, a feeling for patients.” X9T3B*

From a systemic perspective, students highlighted that nursing is indispensable for those unable to care for themselves due to illness, age, or vulnerability. Participants emphasized that, given demographic changes and rising long-term care needs, nursing’s significance within healthcare is increasing.

“And I mean, nursing care is ultimately simply for people who can no longer take care of themselves or are in need because, for example, they may have an illness or something like that. So seen from that perspective, they receive a certain kind of care there that they might no longer be able to provide for themselves. And, if you take a look at demographics now, the people are getting older, umm, which means that we need still more nursing staff. This means that it is also highly relevant in this regard.” X9T3B

Finally, some students reported a positive shift in their perception of nursing during training. They valued supportive ward encounters and collegial interactions, while noting that poor communication and teamwork can undermine patient care, highlighting the importance of respectful interprofessional collaboration.

#### Patient well-being

Students discussed patient well-being as a central outcome of interprofessional collaboration and nursing care. Within this category, the following subcategories emerged.

- Teamwork and patient well-being
- Patient-centered care

Students consistently emphasized that patient well-being depends on the quality of interprofessional teamwork, noting that failed collaboration or fragmented communication can lead to information loss and ultimately compromise patient care.

*“I believe that if the patient feels quasi-poorly cared for, then it is also harder for the body to become healthy again. Thus, I would say that if people are located in an environment where they are treated well, and, above all, also well cared for […]” G2M7Y*

Participants emphasized that effective cooperation enables teams to respond more adequately to patients’ needs, fostering a safer and more supportive clinical environment. Beyond structural collaboration, students described nursing as inherently patient-centered, highlighting empathy, human closeness, and sensitivity to subtle changes as essential for recovery and respectful care.

## Discussion

### Main findings

This qualitative study aimed to explore how German medical students perceive nursing roles, nursing-related learning experiences, and interprofessional collaboration within undergraduate medical education. Using semi-structured interviews and Mayring’s qualitative content analysis, seven themes were identified: Interprofessional Collaboration and Role Understanding; First Clinical Experiences; Nursing During the Practicum; Nursing-Related learning experiences During Medical School; Desired Nursing -related Learning Experiences; PImportance of Nursing-related content; and Patient Well-being.

Across these themes, students emphasized that interprofessional collaboration and mutual role understanding between physicians and nurses are essential for effective patient care (13). Consistent with the categories “Interprofessional Collaboration” and “Patient Well-being,” participants described both the importance of teamwork and the negative consequences of insufficient communication for clinical practice. Students explicitly linked communication failures to delayed information exchange, reduced coordination between professional groups, and potentially compromised patient care, thereby reinforcing the relevance of interprofessional collaboration for patient-centered outcomes.

The mandatory nursing internship emerged as the most formative influence on these perceptions. While students valued early clinical exposure and basic skills acquisition, they reported frustration with limited structure, supervision, and clarity.

#### Early clinical experiences and professional identity formation

Taken together, these findings demonstrate that students’ early clinical experiences, perceptions of nursing roles, nursing-related practical skills, clinical workflows, and nursing-related learning experiences are closely intertwined with their understanding of interprofessional collaboration and its relevance for patient-centered care. These findings align with theories of professional identity formation, which conceptualize workplace learning and socialization as central to the development of professional values and roles among medical trainees (14). Recent theoretical and empirical work on Extended Professional Identity Theory (EPIT) similarly emphasizes that interprofessional identity formation develops through repeated workplace-based interactions and socialization processes within clinical learning environments (15).

In this context, the nursing internship may function as a hidden curriculum shaping interprofessional perceptions through implicit norms, role modeling, and everyday clinical interactions beyond formal teaching structures (16). The results of this study further suggest that nursing-related experiences are integrated into students’ emerging understanding of clinical practice and interprofessional collaboration rather than being perceived as separate from medical training.

Previous studies confirm the limited and somewhat stereotypical understanding of nursing roles among medical students. Eich-Krohm et al. (17) demonstrated that the nursing practicum can enhance interprofessional awareness, but its impact remains inconsistent without clear educational framing. Other empirical studies have shown that structured interprofessional education (IPE) positively influences medical students’ perceptions of nursing. Lee et al. (18) demonstrated that simulation-based IPE significantly improves understanding and appreciation of nursing roles, thereby fostering more collaborative attitudes. Similarly, Spaulding et al. (3) showed that structured IPE enhances interprofessional readiness for collaboration.

However, such approaches remain underdeveloped in German medical curricula, particularly regarding nursing. Despite policy-level recognition of IPE’s importance in Germany (8, 9), empirical evidence on medical students’ perceptions and experiences of nursing is limited (19).

Students consistently identified the nursing internship as their primary exposure to nursing and described it as highly salient despite its marginal curricular status. Similar findings have been described in international studies on practical nursing placements for medical students, which suggest that early immersion in nursing practice contributes to role understanding and interprofessional socialization, but depends strongly on structured supervision and educational framing (20).

While patient contact and skill acquisition were valued, many felt underused, particularly when poorly integrated into care teams or lacking supervision. These findings suggest that informal workplace learning dominates this phase, potentially leading to inconsistent learning experiences and role understanding.

Such unstructured internships may limit professional role understanding, with potential downstream effects on interprofessional collaboration, clinical workflows, and patient safety.

### Implications for interprofessional education

Previous studies by the authors showed that in early clerkships, the absence of defined learning objectives leads students to set their own goals, often focusing on practical skills and clinical exposure (21). Similarly, highly structured and engaging learning formats, such as subject-specific international electives, were shown to promote reflection and disciplinary interest (22). In line with these findings, a clearly structured nursing rotation with defined clinical learning objectives may enhance educational outcomes. Highlighting distinct responsibilities counters the common reduction of nursing to basic bedside tasks (e.g., bedding, toileting, feeding) and fosters a deeper appreciation of interprofessional roles. Given the increasing ongoing academization and professionalization of nursing in Germany, it is essential that medical students gain insight into nurses’ professional clinical, organizational, and documentation responsibilities (23).

Structured learning opportunities should therefore promote understanding of these evolving roles and strengthen interprofessional role clarification within undergraduate medical education.

This aligns with students’ expectations for nursing-related learning experiences, including structured teaching of basic clinical skills, patient-centered communication, and management of vulnerable patient groups. These findings indicate a need for structured curricular integration that combines practical training with opportunities for interprofessional learning and reflection.

The frequent perception of the internship being “too long” likely reflects frustration with insufficient structure rather than content overload. Addressing this through IPE may strengthen interprofessional understanding and communication by engaging students in collaborative, inquiry-driven activities and promoting the longitudinal integration of nursing within medical curricula (24). Importantly, students linked the quality of interprofessional collaboration and nursing care to patient well-being, emphasizing that fragmented communication and poor teamwork may negatively affect patients’ recovery, safety, and perceived quality of care (25).

Furthermore, fostering research-oriented interprofessional learning environments early in training may support shared understanding of each profession’s scientific and clinical contributions and could drive innovation in addressing current healthcare system challenges (23, 26).

The present findings highlight the educational potential of the nursing internship as one of students’ first encounters with nursing practice and interprofessional care. Because it is often completed before formal medical training, structured educational support is needed to maximize its value for interprofessional education (IPE).

Across nursing internships, clerkships, and the PJ, insufficiently defined learning objectives and limited longitudinal integration reduce opportunities for progressive development of interprofessional competencies (21).

As a result, students have limited opportunities to reflect on their evolving professional roles and interprofessional role understanding, potentially limiting their preparedness for collaborative clinical practice.

### Strengths and limitations

The strengths of this study include the selection of participants across all academic years, enabling insights into different stages of professional development. In addition, the credibility and trustworthiness of the findings were strengthened through the use of established qualitative methods, independent coding by multiple researchers, investigator triangulation, and interdisciplinary validation of the category system. These procedures enhanced the rigor of data interpretation and supported the credibility and transferability of the findings.

Several limitations should also be considered. The small, voluntarily recruited sample may introduce selection bias and may limit the transferability of findings. However, iterative analysis indicated substantial thematic sufficiency after approximately 12 interviews, with subsequent interviews contributing primarily confirmatory rather than novel insights. Although some subthemes were supported by comparatively few participants and should therefore be interpreted as reflecting the diversity of students’ perspectives rather than their prevalence, the overarching themes were consistently supported across the dataset. Additional representative quotations for each main category are provided in Supplementary Material 2 to further enhance transparency.

Although participants were drawn from multiple institutions and study years, no formal stratification was performed. The peer-to-peer interview setting may have increased social desirability bias. In addition, experiences of the nursing practicum varied across clinical contexts, reducing comparability. The reliance on retrospective self-reporting introduces potential recall bias. Finally, participant validation was not conducted, which may affect the confirmability of the findings. To mitigate this limitation, analytical rigor was strengthened through investigator triangulation, including independent coding and interdisciplinary validation in a research workshop. Future qualitative studies may further reduce social desirability bias by employing interviewers who are independent of the participants’ peer group, incorporating anonymous written reflections, or recruiting participants across multiple institutions to broaden perspectives and reduce potential influences of shared educational cultures. As with all qualitative studies, the findings are intended to provide analytical rather than statistical generalization.

### Future directions

Although conducted in Germany, the identified challenges–unstructured early clinical exposure, limited interprofessional role clarity, and insufficient curricular integration of nursing–mirror issues reported internationally in early IPE exposure (6, 8).

This suggests that insufficiently structured early clinical experiences and limited interprofessional role understanding are common challenges across healthcare education systems with comparable training structures. Future research should include multisite quantitative studies and pilot implementations of structured interprofessional modules to evaluate their potential effects on collaborative practice, role understanding, and patient-centered outcomes, thereby informing future curriculum development in medical education. This may pave the way for the German medical school curriculum to move beyond framing nursing solely as practical care and instead integrate nursing roles, nursing-related practical skills, clinical workflows, nursing-related learning experiences, and perspectives more systematically into interprofessional medical training. Accordingly, early nursing placements should not merely function as observational exposure, but as structured interprofessional learning environments that actively support professional identity formation and collaborative competence development.

## Conclusion

The findings of this study demonstrate that German medical students’ perceptions of nursing roles, nursing-related learning experiences, and interprofessional collaboration and Role Understanding are strongly shaped by early clinical experiences, particularly the mandatory nursing internship. While students regarded interprofessional collaboration as essential for patient-centered care, many reported uncertainty regarding nursing roles and responsibilities and described considerable variability in the educational value of their nursing placements. Beyond the nursing internship, targeted IPE units should be embedded in major clinical rotations such as Internal Medicine and Surgery, with clear objectives linking nursing science to each discipline based on the present findings. Such an approach may support the development of interprofessional role understanding, collaborative practice, role clarity, and patient-centered care. A longitudinal integration across clinical training may further enable students may enable students to gain early nursing experience while progressively reflecting on and consolidating their interprofessional role understanding throughout medical training.

## Data Availability

The original contributions presented in this study are included in this article/Supplementary material. Further inquiries can be directed to the corresponding author.
