# Supplementary material for: Medical students’ perception of the role of nursing in medical care: implications for interprofessional education
Source: Front Med (Lausanne). 2026 Aug 10;13:1877755. doi: 10.3389/fmed.2026.1877755 (PMC13501579; doi:10.3389/fmed.2026.1877755)
Supplement: Supplementary file 2 [file Table_1.docx]

| **Supplemental Material 1.** Interview guide used for medical students’ interviews. | |
| --- | --- |
|  |  |
| **English translation** | **German original** |
|  |  |
| 1. In your opinion, what significance does nursing have in medical education? | 1. Welche Bedeutung hat die Pflege Ihrer Meinung nach in der medizinischen Ausbildung? |
| 2. In your opinion, what significance does nursing have for you personally? | 2. Welche Bedeutung hat die Pflege Ihrer Meinung nach für Sie selbst? |
| 3. In your opinion, what is the general significance of nursing care? | 3. Welche Bedeutung hat die Pflege Ihrer Meinung nach generell? |
| 4. How do you regard the representation of nursing in medical education? | 4. Wie schätzen Sie die Repräsentation in Pflege in der medizinischen Ausbildung ein? |
| 5. In your opinion, what are the most appropriate formats and times for placement of nursing content during the course of study? | 5. Was sind Ihrer Meinung nach geeignete Formate und Zeitpunkte zur Vermittlung pflegerischer Inhalte im Studium? |
| 6. In your opinion, which nursing topics are relevant for medical school studies? | 6. Welche pflegerischen Inhalte sind Ihrer Meinung nach relevant für das Medizinstudium? |
| 7. What requests and suggestions do you have regarding the teaching of nursing content during medical school studies? | 7. Welche Wünsche und Vorstellungen haben Sie zur Vermittlung pflegerischer Inhalte im Studium? |
