# Supplementary material for: Medical students’ perception of the role of nursing in medical care: implications for interprofessional education
Source: Front Med (Lausanne). 2026 Aug 10;13:1877755. doi: 10.3389/fmed.2026.1877755 (PMC13501579; doi:10.3389/fmed.2026.1877755)
Supplement: Supplementary file 3 [file Table_2.docx]

| **Supplemental Table 2.** Categories, themes and anchor quotations derived from the medical students interviews on nursing. | | | |
| --- | --- | --- | --- |
|  |  |  |  |
| **English translation** | | **German original** | |
|  |  |  |  |
| **Interprofessional Collaboration** | | **Interprofessionelle Zusammenarbeit** | |
| 1.1 Communication and understanding between the professions | „OK, well, in principle, one must understand what the other working groups do all day long and where their areas of responsibility lie. [...] At the same time, communication is something one also has to learn in a somewhat practical way, and you can also gladly look at all sides of it.*“ 1CJYR* | Kommunikation und Verständnis zwischen den Professionen | „Okay, naja, also prinzipiell muss man ja verstehen, was die anderen Arbeitsgruppen so einen ganzen lieben langen Tag machen, wo welche Zuständigkeitsbereiche sind. […] Gleichzeitig Kommunikation muss man natürlich so ein bisschen auch praxisnah lernen und da kann man sich auch gerne mal alle Seiten angucken.“ *1CJYR* |
| 1.2 Interprofessional cooperation: Perceived attitude and respect felt | “Well, without nursing, nothing would really work in the hospital, and yes, I also find that nursing is always underappreciated there, for example.” *UR6P1* | Interprofessionelle Miteinander - Wahrgenommene Haltung und empfundener Respekt | „Also ohne die Pflege wird halt gar nichts laufen im Krankenhaus und ja ich finde auch, dass die Pflege da auch immer zu kurz kommt von der Wertschätzung her z.B. auch.“ *UR6P1* |
| 1.3 Experiences working together with nursing staff in everyday clinical practice | "I also think that the cooperation doesn’t always run optimally; that's the impression I have as well. But except for the three months of the nursing practicum in the pre-clinical phase, where one really still doesn’t actually have anything to do with the clinic, I think that one could do more or emphasize more what the role of nursing is, and not always separate it so that one says, 'Yes, that's what the nurses do, that's what the doctors do,' because one could perhaps try to collaborate more." *PZZ7V* | Erfahrungen mit der Zusammenarbeit mit der Pflege im klinischen Alltag | „Ich glaube auch die Zusammenarbeit läuft nicht immer gerade optimal, habe ich so einen Eindruck auch. Aber abgesehen von den drei Monaten Pflegepraktikum in der Vorklinik, wo man eh auch mit der Klinik eigentlich noch nichts zu tun hat, finde ich, könnte man, glaube ich, mehr machen oder mehr darauf hinweisen, was so die Rolle der Pflege ist und das nicht immer so auftrennen, dass man dann sagt, ja, das machen die Schwestern, das machen die Ärzte, weil man könnte da mehr vielleicht versuchen zusammenzuarbeiten.“ *PZZ7V* |
| 1.4 Structural aspects of interprofessional collaboration | „[…] because in many hospitals, the nursing staff does that too, but in some cases not. And then it would be really good if you could do that which is actually expected.“ *JXJ1N* | Strukturelle Aspekte interprofessioneller Zusammenarbeit | „„[…] weil das ist ja in vielen Häusern macht das auch die Pflege, aber teilweise ja auch nicht. Und dann wäre es ganz gut, wenn man sowas kann, was ja eigentlich vorausgesetzt wird.“ *JXJ1N* |
| 1.5 Individually ascribed meaning of interprofessional collaboration | „OK, so I think it's really important because collegial cooperation can only work if you also have some knowledge of it and also get involved in that area, especially when you have contact during an internship or something like that." *JXJ1N* | Individuell zugeschriebene Bedeutung interprofessioneller Zusammenarbeit | „OK, also ich finde es an sich super wichtig, weil die kollegiale Zusammenarbeit nur funktionieren kann, wenn man da auch ein bisschen Bescheid weiß und sich auch engagiert in die Richtung, wenn man dann im Praktikum oder so mal Kontakt hat.“ *JXJ1N* |

| **2.First clinical experiences** | | **Erste Erfahrungen im Krankenhaus** | |
| --- | --- | --- | --- |
| 2.1 First practical experiences and contact with daily clinical practice | "I'm just starting with that now. Yes, as I already said, so if you have never been to a hospital before, or worked in the medical field in general, that's where you really first learn the basics. Thus, you learn how to deal with people, how to talk to people, how to talk to patients, how to deal with patient histories, and you also come into contact for the first time with sick people or care-dependent people.” *X9T3B* | Erste Praxiserfahrungen und Kontakt zum klinischen Alltag | „Da bin ich ja jetzt gerade erst drin. Ja, was ich schon gesagt habe, also wenn man vorher noch nie im Krankenhaus war, oder allgemein im medizinischen Bereich gearbeitet hat, lernt man dort halt erstmal wirklich die Grundlagen. Also man lernt, wie man mit Leuten umgeht, wie man mit Leuten redet, wie man mit Patienten redet, wie man mit Patientengeschichten umgeht, man kommt auch das erste Mal mit kranken Menschen oder pflegebedürftigen Menschen in Kontakt.“ *X9T3B* |
| 2.2 Subjective experience of the nursing practicum | “Well, it was a whole lot. As I said, I think it was also simply because I was in two pediatric wards, one each month, and you just can't do much with children, you're not allowed to. And unfortunately, it often came down somehow to cleaning and making beds and so on. These low-level tasks didn't add any value for me." *20VFF* | Subjektives Erleben des Pflegepraktikums | „Also es war sehr, sehr viel. Also wie gesagt, ich glaube, es lag auch einfach viel daran, dass ich einfach auf zwei Kinderstationen war, jeweils im Monat und du bei Kindern nun mal einfach nicht viel machen kannst, machen darfst. Und da lief es halt leider doch oft darauf hinaus, das irgendwie so zu putzen musste und Betten zu beziehen und so. Diese Billotätigkeiten, das hat mir halt keinen Mehrwert gebracht.“ *20VFF* |
| 2.3 Structure and preparation of the nursing practicum | “Very exhausting. So, I did not feel very appreciated as I found three months of unpaid work during the holidays a lot. So I think less would have been enough, and more appreciative interactions in return.” *GPR6C* | Struktur und Vorbereitung des Pflegepraktikums | “Sehr anstrengend. Also ich habe mich nicht so wertgeschätzt gefühlt, weil halt drei Monate in den Ferien unbezahlt fand ich viel. Also ich glaube weniger hätte gereicht und dafür so wertschätzenderer Umgang.“ GPR6C |
| 2.4 Patient contact and practical activities in the context of nursing | “That was also the case with us, we somehow started in the beginning with learning some basic skills, like how to make a bed and how to properly brush a patient's teeth and so on. You could argue about the content we covered, but I thought it was good." *20VFF* | Patientenkontakt und praktische Tätigkeiten im Pflegekontext | *„*Das war ja auch mit dem, wir haben am Anfang irgendwie so ein bisschen so Basic Fähigkeiten, wie man ein Bett bezieht und wie man einem Patienten richtig die Zähne putzt und so. Über die Inhalte kann man jetzt streiten, die wir da gemacht haben. Aber das fand ich schon gut.“ 20VFF |
|  |  |  |  |
| **3.Nursing During the Practicum** | | **Pflege im Praktikum** | |
| 3.1 Personal perception of nursing as a professional group in the context of the practicum | “I found, for example, that where I really noticed this the first time was naturally during the nursing practicum, which is sort of the first thing you experience when you start your studies. I think it’s super important to have contacts there, simply to get to know the other side a bit at first. Also, the appreciation that is shown to nursing staff. And what also really struck me was that the nurses naturally see patients for much longer than the doctors do. And I have very often noticed that there were moments when I thought to myself afterwards that one should have listened to the nurse sooner or asked earlier, then one could have- I don't know, prevented this and that." *BJ1V6* | Wahrnehmung und Erfahrungen im Pflegepraktikum | “Ich fand z.B. wo ich das erste Mal sehr stark mitbekommen habe, natürlich Pflegepraktikum ist ja irgendwie so das erste, was man so mitbekommt, wenn man im Studium angekommen ist. Ich finde es super wichtig, dass man da die Kontakte hat, einfach um so ein bisschen erstmal die andere Seite natürlich kennenzulernen. Auch so Wertschätzung die der Pflege entgegengebracht wird. Und was mir auch krass aufgefallen ist, dass die Pflege sieht den Patienten, die Patientin natürlich meistens deutlich länger als die Ärzte und Ärztinnen das tun. Und mir ist sehr oft aufgefallen, dass es Momente gab, wo ich mir danach gedacht habe, da hätte man jetzt mal eher auf die Pflegekraft hören sollen oder mal früher danach fragen sollen, dann hätte man- keine Ahnung, das und das verhindern können.“ *BJ1V6* |
| 3.2 Supervision, guidance, and mentoring by nursing staff | “The collaboration was actually really nice with both, they showed a lot, I was actually always involved in many things." *UR6P1*  „So not much in terms of specialized skills, a bit like everyday life on the ward. I just did all the work that no one else wanted to do." GPR6C | Betreuung, Anleitung und Mentoring durch Pflegekräfte | “Zusammenarbeit war eigentlich bei beiden echt schön, haben viel gezeigt, war bei vielen Sachen dabei eigentlich immer.“ *UR6P1*  “Also fachlich nicht viel, ein bisschen wie so Stationsalltag. Ich hab halt die ganze Arbeit gemacht, die niemand anders machen wollte.“ *GPR6C* |
| 3.3 Structure and organization of the nursing practicum | “I wish that you were given a little training beforehand on how things work, that you were given an explanation. At the beginning, I just stood around a lot, looking stupid, and I wish that you were not actually scheduled in as an unpaid worker, but that you were shown things and perhaps already know how to do them beforehand, so that the workflow on the ward runs better.“ *GPR6C* | Struktur und Organisation des Pflegepraktikums | „Ich würde mir wünschen, dass man vorher so bisschen angelernt wird, wie das läuft, dass man sowas erklärt bekommt. Am Anfang stand ich viel, einfach dumm rum, und dass man nicht irgendwie so eigentlich als unbezahlte Arbeitskraft so fest mit eingeplant wird, sondern dass man Sachen gezeigt bekommt und vielleicht vorher schon kann, so dass der Ablauf auf Station dann besser läuft.“ *GPR6C* |
|  |  |  |  |
| **4. Nursing-related Learning Experiences During Medical School** | | **Lernerfahrungen zur Pflege im Studium** | |
| 4.1 Curricular integration of nursing content | “Well, I think that medical school teaches you very little about nursing and how to care for patients properly. One has a 90-day nursing practicum in the pre-clinical phase; nevertheless, many uncertainties remain [...] And these are also points that are definitely being addressed more and more thoroughly in nursing training in comparison to medical school.” *POA6W* | **Curriculare Integration pflegerischer Inhalte** | „Also ich finde, im Medizinstudium lernt man eher wenig über die Pflege und wie man Patienten richtig pflegt, man hat ja das Pflegepraktikum von 90 Tagen in der Vorklinik und nichtsdestotrotz bleiben da viele Unklarheiten […] Und das sind auch so Punkte, wo in der pflegerischen Ausbildung bestimmt mehr und mehr darauf eingegangen wird. Im Vergleich zum Medizinstudium.“ POA6W |
| 4.2 Theoretical analysis of nursing | "So in terms of theoretical content, I do not necessarily need to know how to wash a patient, I agree with that, I don't necessarily need that, but maybe a couple of little things, like how to position the patient properly, how to get patients out of bed properly without their sliding halfway down somewhere, because you sometimes have to do that yourself. Sometimes you find yourself in situations where you just have to know these things. And I am of the opinion that's something you should already know." *23OF9* | Theoretische Auseinandersetzung mit Pflege | „Also im Sinn, also theoretische Inhalte, ich muss jetzt nicht unbedingt wissen, wie man den Patienten wäscht, gehe ich mit, das brauche ich nicht unbedingt, aber vielleicht auch so ein paar Kleinigkeiten wie, wie lager ich den Patienten gut, wie kriege ich den raus aus dem Bett ordentlich, ohne dass er mir halb runterrutscht irgendwo, weil du das ja auch manchmal selbst tun musst. Du kommst ja auch manchmal in Situationen, wo du das einfach wissen musst. Und da bin ich der Meinung, das sollte man dann vielleicht auch schon drauf haben.“ *23OF9* |
| 4.3 Practical nursing-related learning during Medical School | "I think that I'm still lacking some very basic skills that nursing staff have, for example, like how to set up an infusion correctly, how to handle a peripheral IV line properly so that it works well and runs smoothly. I didn’t learn that during the nursing practicum. […] But I think it would also somehow be helpful to have an afternoon again or maybe even a block practicum, you know, to integrate that." 20VFF | Praktische Inhalte zur Pflege im Studium | „Ich glaube, mir fehlt es halt auch noch so ganz basic Fähigkeiten, die halt die Pflege kann, wie richte ich eine Infusion z.B., wie gehe ich am besten mit einer Flexüle um, dass die halt schön ist und läuft und so. Das habe ich im Pflegepraktikum nicht gelernt. […] aber ich glaube, dass auch noch mal praktisch irgendwie einfach noch mal Nachmittag wie so ein Blockpraktikum, weißt du, nochmal mit zu integrieren.“ *20VFF* |
| 4.4 Perception of the importance of nursing-related content during medical school | "OK, so I think it's really important because collegial cooperation can only work if you also have some knowledge of it and also get involved in that area, especially when you have contact during a practicum or something like that." *JXJ1N* | Wahrnehmung der Bedeutung pflegerischer Inhalte im Studium | „OK, also ich finde es an sich super wichtig, weil die kollegiale Zusammenarbeit nur funktionieren kann, wenn man da auch ein bisschen Bescheid weiß und sich auch engagiert in die Richtung, wenn man dann im Praktikum oder so mal Kontakt hat.“ *JXJ1N* |
|  |  |  |  |
| **5. Desired nursing-related learning experiences** | | **Gewünschte pflegerische Inhalte** | |
| 5.1 Practical skills and workflows | “I think that a precise, structured way of working [is important] — like, how do I organize certain procedures? How do I ensure the hygienic execution of specific nursing routines?” *POA6W*  *„…I think I'm also still lacking very basic skills that nursing can do, for example, how do I set up an IV, how do I best handle an IV cannula so that it is nice and runs well and everything.“*  „Yeah, to this day I still can't (handle) an IV properly. I learned very little about these basic skills.“ *20VFF*  „So, preparing an IV is something everyone should be able to do, connecting an IV, disconnecting it. I know it took ages until I knew which IV you flush before, which one you don't, after which one, because everyone somehow did it differently.“ GPR6C | Praktische Fertigkeiten und Arbeitsabläufe | „Ich finde so eine präzise, strukturierte Arbeitsweise, also wie strukturiere ich bestimmte Abläufe? Wie achte ich auf die hygienische Ausführung bestimmter Pflegeroutinen?“*POA6W*  „…ich glaube mir fehlt es halt auch noch so ganz basic Fähigkeiten, die halt die Pflege kann, wie richte ich eine Infusion z.B. wie gehe ich am besten mit einer Flexüle um, dass die halt schön ist und läuft und so.“  „Ja, eine Infusion kann ich bis heute nicht richtig. Von diesen basic Fähigkeiten habe ich sehr wenig gelernt.“ *20VFF*  „Also Infusion richten sollte ja jeder können, Infusion anbauen, abbauen. Ich weiß, das hat ewig gedauert bis ich so wusste, vor welcher Infusion spült man jetzt vor welcher nicht, nach welcher, weil alle das irgendwie anders gemacht haben.“ GPR6C |
| 5.2 Patient interaction and communication skills | “And also, if I understood correctly, simply the contact with patients in general.” *POA6W*  *„*Definitely patient contact, how do I handle people well?“ *BJ1V6*  „How can I facilitate communication between nursing and doctors?“ 23OF9 | Patientenumgang und kommunikative Kompetenz | „Und halt auch, wenn ich richtig verstanden habe, auch so dieser einfach überhaupt der Kontakt mit Patienten.“ POA6W  „Auf jeden Fall Patientenkontakt, wie gehe ich mit den Leuten gut um? BJ1V6“  „Wie kann ich die Kommunikation zwischen der Pflege und den Ärzten erleichtern?“ 23OF9 |
| 5.3 Dealing with special patient groups and stressful situations | "And also what are some conflict situations that can arise in everyday life with patients? How do I deal with certain patient groups where it's difficult to follow the usual nursing routine, for example, in cases of dementia, or if patients could potentially be aggressive, or when they can’t engage, or even when they’re not capable of giving consent? And how do you handle this, so that even the more difficult cases are taken into consideration, also these conflict resolution skills [are needed]!" *POA6W* | Umgang mit besonderen Patientengruppen und Belastungssituationen | „Und auch so was sind so Konfliktsituationen, die entstehen können im Alltag mit Patientinnen und Patienten? Wie gehe ich mit bestimmten Patientengruppen um, wo es schwer ist, der pflegerischen Routine nachzugehen, z.B. bei Demenz oder wenn Patienten ein Aggressionspotenzial haben oder wenn sie sich darauf nicht einlassen können oder sogar, wenn sie nicht einwilligungsfähig sind? Und wie macht man das, sodass auf schwierigere Fälle auch eingegangen wird, auch diese Konfliktlösungskompetenz!“ *POA6W* |
| 5.4 Nursing-related foundational knowledge | „Well, in principle, one must understand what the other working groups do all day long and where their areas of responsibility lie.“ *1CJYR*  „One could also argue that there should be a lot of basic care…“ JXJ1N | Pflegebezogenes Hintergrundwissen | „Also prinzipiell muss man ja verstehen, was die anderen Arbeitsgruppen so einen ganzen lieben langen Tag machen, wo welche Zuständigkeitsbereiche sind.“ *1CJYR*  „Man könnte auch argumentieren, dass es viel Grundpflege geben sollte…“ JXJ1N |

| **6. Importance of nursing** | | **Bedeutung der Pflege** | | |
| --- | --- | --- | --- | --- |
| 6.1 Personal significance | ”I actually think nursing is quite important, within the framework of the studies, for teaching these basics like I just said, but also nursing imparts somewhere an element of human closeness, I would simply say. So, there’s also a big part of empathy involved. Obviously, you don't learn all the medical stuff, like the concrete medical things, but you do learn, or you just simply somehow develop there, a feeling for patients.” *X9T3B* | Persönliche Bedeutung | | “Ich finde Pflege tatsächlich sogar ziemlich wichtig , also im Rahmen des Studiums, zur Vermittlung dieser Grundlagen, die ich gerade gesagt habe, aber Pflege vermittelt halt irgendwo auch eine gewisse Menschennähe, würde ich einfach sagen. Also da ist ja auch ein großer Teil von Empathie mit bei. Klar, du lernst halt nicht die ganzen medizinischen, also die konkreten medizinischen Sachen, aber du lernst halt, oder du entwickelst dort halt auch einfach irgendwie ein Gefühl für Patienten.“ *X9T3B* |
| 6.2 Systemic significance | ”And I mean, nursing care is ultimately simply for people who a) can no longer take care of themselves or are in need because, for example, they may have an illness or something like that. So seen from that perspective, they receive a certain kind of care there that they might no longer be able to provide for themselves. And, if you take a look at demographics now, the people are getting older, umm, which means that we need still more nursing staff. This means that it is also highly relevant in this regard." *X9T3B* | Systemische Bedeutung | | „Und ich meine, Pflege ist ja letztendlich einfach für Leute, die sich a) nicht mehr um sich selbst kümmern können oder halt bedürftig sind, weil sie zum Beispiel vielleicht eine Krankheit haben oder sowas, halt so gesehen die Perspektive, dass sie dort halt eine gewisse Art von Versorgung bekommen, die sie sich selber vielleicht nicht mehr gewährleisten können. Und, wenn man sich jetzt mal die Demographie anguckt, die Leute werden immer älter, ähm, heißt, wir brauchen noch mehr Pflege. Heißt, die hat auch in der Hinsicht eine große Relevanz.“ *X9T3B* |
| 6.3 Change in the perception of nursing | “Well, I have very positive memories of it because I was in nice wards, [...] I was well received, I would say. And yes, I liked it, I also thought it was good that I did it, I had to anyway, but I also found it a positive experience for me.” *1NH5F* | Veränderung des Pflegebildes | | „Also ich hatte, ich habe eine sehr positive Erinnerung daran, weil ich auf netten Stationen war, […] ich wurde gut aufgenommen, würde ich sagen. Und ja, also mir hat es gefallen, ich fand es auch gut, dass ich es gemacht habe, also musste ich ja eh, aber ich fand es für mich auch eine positive Erfahrung schön.“ *1NH5F* |
| **Patient Well-being** | | **Patientenwohl** | | |
| 7.1 Teamwork and patient well-being | I believe that if the patient feels quasi-poorly cared for, then it is also harder for the body to become healthy again. Thus, I would say that if people are located in an environment where they are treated well, and, above all, also well cared for […]“*G2M7Y* | Teamarbeit und Patientenwohl | | „Ich glaube, wenn der Patient sich quasi schlecht gepflegt fühlt, dann ist es auch schwerer für den Körper wieder gesund zu werden. Von daher würde ich sagen, dass, wenn die Menschen sich in einem Umfeld befinden, wo sie gut behandelt werden und vor allem auch gut gepflegt werden […]“*G2M7Y* |
| 7.2 Patient-centered care | *”… but nursing imparts somehow also an element of human closeness, I would simply say. So, there’s also a big part of empathy involved….”* *X9T3B* | Patientenorientierung | | „ …aber Pflege vermittelt halt irgendwo auch eine gewisse Menschennähe, würde ich einfach sagen. Also da ist ja auch ein großer Teil von Empathie“ *X9T3B* |
|  |  |  | |  |
|  |  |  | |  |
